# Expression of Concern: Facemask-wearing behavior to prevent COVID-19 and associated factors among public and private bank workers in Ethiopia

**DOI:** 10.1371/journal.pone.0327826

**Published:** 2025-07-08

**Authors:** The PLOS One Editors

The *PLOS One* Editors issue an Expression of Concern to alert readers that this article [1] was identified as one of a series of submissions for which we have concerns about authorship and adherence to the journal’s first and second publication criteria. We regret that these issues were not addressed prior to the article’s publication. In addition, the article [1] appears to report the same study and results as an article published later by the same author group [2].
