# Supplementary material for: Facemask-wearing behavior to prevent COVID-19 and associated factors among public and private bank workers in Ethiopia
Source: PLoS One. 2021 Dec 1;16(12):e0259659. doi: 10.1371/journal.pone.0259659 (PMC8635365; doi:10.1371/journal.pone.0259659)
Supplement: S1 File — (DOCX) [file pone.0259659.s001.docx]

# Name and signature of data collector: - ________________________ Date _____________

Name and signature of supervisor: - ________________________

| Questionnaire number | _____________________ |
| --- | --- |
| Bank branch | 1. Government bank 2. Private bank |
| Part 1: Socio-demographic status | |
| 1. Sex of respondent | 1. Male 2. Female |
| 1. Age of respondent (years) | _____________________ |
| 1. Education level | _____________________ |
| 1. Monthly income (ETB) |  |
| 1. Marital status | - - - 1. Single 3. Divorced       2. Married 4. Widowed |
| 1. Religion | Muslim 3. Protestant  Orthodox 4. Other/ specify______________ |
| 1. Ethnicity | _______________________ |
| 1. Experience in the bank (years) | _______________________ |
| 1. Position as cashier (days/week) | _______________________ |
| 1. Place of residence | - - - 1. Urban 2. Rural |
| 1. Family size (persons) | _______________________ |
| 1. Have children in household | 0. No 1. Yes |
| 1. Have family members >65 years old in household | 0. No 1. Yes |
| 1. No. of staff in the bank | _________________________ |
| 1. No. of customers of the bank (days) | _________________________ |

**Part 2: - Knowledge on** COVID**-19 related question**

| **Items** | **Yes** | **No** | **Don’t know** |
| --- | --- | --- | --- |
| 1. COVID-19 is caused by virus |  |  |  |
| 1. The signs and symptoms of COVID-19 include fever, fatigue, dry cough and shortness of breath. |  |  |  |
| 1. Unlike the common cold, stuffy nose, runny nose, and sneezing are less common in persons infected with the COVID-19 virus. |  |  |  |
| 1. Currently there is no vaccine available for COVID-19 in Ethiopia. |  |  |  |
| 1. There is no effective curative treatment for COVID-19. |  |  |  |
| 1. Only those who are older age or having underlying disease are at a high risk of deterioration into serious condition. |  |  |  |
| 1. The main mode of COVID-19 transmission is through contact. |  |  |  |
| 1. People without symptoms can still transmit COVID-19 to others. |  |  |  |
| 1. Persons with COVID-19 cannot infect/ spread the virus to others when a fever is not present. |  |  |  |
| 1. Eating or contacting wild animals would result in the infection by the covid-19 virus. |  |  |  |
| 1. The time from exposure to onset of symptoms is 2-14 days. |  |  |  |
| 1. Mask wearing prevent infection by the COVID-19 virus |  |  |  |
| 1. Isolation and treatment of people who are infected with the COVID-19 virus are effective ways to reduce the spread of the virus. |  |  |  |
| 1. Proper washing hand for at-least 20 seconds with soap and water is one method of preventing COVID-19. |  |  |  |
| 1. There is a need to wash hands before and after toughing all things outside your home. |  |  |  |
| 1. Being 2 meters apart from individuals can reduce the risk of transmission of COVID-19 |  |  |  |

**Part 3: Attitude on** COVID **-19 related question**

| **Items** | **Strongly disagree** | **Disagree** | **Neutral** | **Agree** | **Strongly agree** |
| --- | --- | --- | --- | --- | --- |
| - - - 1. COVID-19 is a serious disease. |  |  |  |  |  |
| - - - 1. Wearing a well-fitting facemask are effective in preventing COVID-19 virus. |  |  |  |  |  |
| - - - 1. Hand washing can prevent from COVID-19 virus |  |  |  |  |  |
| - - - 1. COVID-19 is not stigma and I should not hide my infection |  |  |  |  |  |
| - - - 1. Self-efficacies is mandatory to control COVID-19 |  |  |  |  |  |
| - - - 1. I believe I can protect myself against COVID-19 |  |  |  |  |  |
| - - - 1. There is personal risk of being infected with COVID-19 while going to crowded place and meeting with many peoples |  |  |  |  |  |
| - - - 1. I will not go to hospital even if i get sick because of risk of getting infected with covid-19 |  |  |  |  |  |
| - - - 1. COVID-19 can finally be successfully controlled. |  |  |  |  |  |
| - - - 1. If there is an available lab test for detection of the virus, I am willing to do it |  |  |  |  |  |
| - - - 1. If there is an available vaccine for the virus, I am willing to get it |  |  |  |  |  |

| **Part 4: Mask wearing behavior related questions** |  | |
| --- | --- | --- |
| 1. Does the respondent wear a facemask at the time of interview? (Observational) | 0. No 1. Yes | |
| 1. Which part of the face does the worn facemask cover? (Observational) | 1. 1. Nose and mouth 2. 2. Nose, mouth, lower jaw & facial hair 3. 3. Other (Specify) ________ | |
| 3. How often do you wear a mask when you are at work? | A. Always  B. Sometimes  C. Never | |
| 4. What kind of mask do you choose? | A. Surgical/medical facemasks  B. Cloth facemasks  C. N95 facemask  D. Other (Specify) _________ | |
| 5. How often do you change your mask? | 1. for 2-4hrs  2. 1days  3. 2-5 days  4. more than 5days  5. Do not replace the new mask, and continue to use it after cleaning | |
| 6. What is the correct operation for wearing a surgical mask? (Distinguish the inside and outside of the mask, as well as the top and bottom) | | |
| - 1. The mask is light inside and dark outside, and there are metal strips (nose clips) on the top | 0. No 1. Yes | |
| - 1. Fully unfold the folded face of the mask to cover the nose, mouth and chin | 0. No 1. Yes | |
| - 1. Put the fingertips of both hands on the nose clip, start from the middle position, and shape the nose clip according to the shape of the nose bridge | 0. No 1. Yes | |
| - 1. Check whether the edge of the mask fits your face | 0. No 1. Yes | |
| - 1. Wear it regardless of the front and back | 0. No 1. Yes | |
| - 1. Never wear surgical mask | 0. No 1. Yes | |
| 7. After wearing the mask, which of the following behaviors are appropriate? more than one answer is possible) | 1.Touch the front of the mask with your hand  2.Feel free to take off the mask of yourself or others  3.Take off the mask and put it back on  4.Pull the mask down to reveal the nose or mouth  5.None of the above behaviors | |
| 1. Is it comfortable to wear face mask at your work? | 0. No 1. Yes | |
| 1. Does wearing face mask interfere communication? | 0. No 1. Yes | |
| 1. Does face mask leave marks on your faces | 0. No 1. Yes | |
| 1. How do you keep the cleanliness of the facemask? (only for reusable facemasks) | 1. Washing with water 2. Washing with hot water and soap and drying in hot sun for 5 hours 3. Washing and boiling for 5 minutes and drying 4. Ironing the mask for 5 minutes daily 5. Other (Specify)___________ | |
| 1. Where do you collect facemask after used? (observation) | 1. Pedal-operated waste bins 2. Waste container with cover 3. Open waste container without cover 4. Other (Specify)___________ | |
| 1. How is collected waste disposed? | 1. Open disposal 2. Burning 3. Taken by municipality 4. Other (Specify)___________ | |
| **Part 5: Factors related questions for behaviors of mask wearing** | | |
| 1. Do you feel as you are vulnerable to contracting COVID-19? | | 0. No 1. Yes |
| 2. Do you fear of COVID-19? | | 0. No 1. Yes |
| 1. Do you perceive as the consequence of getting COVID-19 is serious? | | 0. No 1. Yes |
| 1. Do you know someone who had test results positive for COVID-19? | | 0. No 1. Yes |
| 1. Do you know someone who was died from COVID-19? | | 0. No 1. Yes |
| 1. Do you have outgoing history or traveled outside the country? | | 0. No 1. Yes |
| 1. What mechanism you used while counting money | | 1.Using saliva  2. Using chemicals  3. Using water  4.Others­­­­­­­­­­­­­­­­­­________________ |
| 1. If your answer for the above question is using saliva, is there any contact of fingers with your tongue? (hand-to perioral routes in the transmission of COVID -19) | | 0. No 1. Yes |
| 1. Do you keep physical distancing of 6 feet (2meters) from coworkers? | | 0. No 1. Yes |
| 1. Do you keep physical distancing of 6feet (2meters) from customer? | | 0. No 1. Yes |
| 11. Cigarette smoking | | 0. No 1. Yes |
| 12. Drinking alcohol | | 0. No 1. Yes |
| 1. Presence of respiratory conditions like sinusitis | | 0. No 1. Yes |
| 1. Presence of chronic illness like hypertension, diabetes | | 0. No 1. Yes |
| 1. Health information dissemination about COVID-19 | | 0. No 1. Yes |
| 16. If yes, from where do you receive information? | | 1.News media e.g., TV**,** radio, newspaper  2. Health care providers  3. Social media  4. Friends  5. Other (Specify)__________ |
| 17. Presence of poster showing mask wearing and hand washing behavior (observational) | | 0. No 1. Yes |
| 18. Do you get training on COVID-19? | | 0. No 1. Yes |

**Thank you for your participation!!!**
