# Supplementary material for: Facemask-wearing behavior to prevent COVID-19 and associated factors among public and private bank workers in Ethiopia
Source: PLoS One. 2021 Dec 1;16(12):e0259659. doi: 10.1371/journal.pone.0259659 (PMC8635365; doi:10.1371/journal.pone.0259659)
Supplement: S2 File — (DOCX) [file pone.0259659.s002.docx]

የመረጃ ሰብሳቢው ስም እና ፊርማ ______________________ ቀን­­­­­­­­­­­­­ _______________

| መጠይቅ ቁጥር |  |
| --- | --- |
| የባንክ ቅርንጫፍ | 1. የመንግስት ባንክ 2. የግል ባንክ |
| ክፍል 1፡ ማህበራዊና ኢኮኖሚያዊ ሁኔታዎች | |
| 1. የመረጃ ሰጪ ፆታ | 1.ወንድ 2. ሴት |
| 1. የመረጃ ሰጪ ዕድሜ(በዓመት) | _____________________ |
| 1. የትምህርት ሁኔታ | _____________________ |
| 1. ወርሃዊ የገቢ መጠን (በብር) | _____________________ |
| 1. ጋብቻ ሁኔታ | - - - 1. ያላገባ 3. የተፋታ/ች       2. ያገባ 4. በሞት የተለየ/ች |
| 1. ሀይማኖት | ሙስሊም 3. ፕሮቴስታንት   1. ኦርቶዶክስ 4. ሌላ (ይገለጽ) ­­--------- |
| 1. ብሄር | _____________________ |
| 1. ባንክ ዉስጥ ያለወት የሥራ ልምድ (በዓመት) | _____________________ |
| 1. በመስኮት (እንደ ገንዘብ ተቀባይ) ስንት ቀን ያገለግላሉ? (በሳምንት) | _____________________ |
| 10. የመኖርያ አድራሻ? | 1. ከተማ 2. ገጠር |
| 1. የቤተሰብ ብዛት | _____________________ |
| 12. በቤት ዉስጥ ልጆች አሉህ/ሽ? | 0. የለም 1. አዎ |
| 13. ቤት ዉስጥ ከ 65 ዓመት በላይ የ ቤተሰብ አባል አለ? | 0. የለም 1. አዎ |
| 14. በባንክ ዉስጥ የሰራተኞች ብዛት | _____________________ |
| 15. አማካይ የደንበኞች ብዛት (በቀን) | _____________________ |

የመረጃ ሰብሳቢዎች ተቆጣጣሪ ስም እና ፊርማ ________________

ክፍል 2: - የጥናቱ ተሳታፊዎችን የኮሮና በሽታ የዕዉቀት ደረጃ ለመለካት የተዘጋጀ መጠይቅ (ትክክለኛዉ መልስ ላይ የ ‘’X’’ ምልክት ይደረግ)

| መጠይቅ | የለም | አዎ | አላዉቅም |
| --- | --- | --- | --- |
| 1. ኮሮና በቫይረስ የሚከሰት በሽታ ነዉ |  |  |  |
| 1. የ ኮሮና ዋነኛ ምልክቶች ትኩሳት ፣ ድካም፣ ደረቅ ሳል እና የትንፋሽ ማጠር ናቸዉ |  |  |  |
| 1. ከጉንፋን በተቃራኒ የአፍንጫ ፈሳሽ፤ የአፍንጫ መዝጋትና ማስነጠስ በኮሮና በሽታ በተያዘ ሰዉ ላይ እምብዛም/ብዙ የተለመደ አይደለም፡፡ |  |  |  |
| 1. በአሁኑ ጊዜ ለ ኮሮና ምንም የተረጋገጠ ክትባት የለም ፡፡ |  |  |  |
| 1. ለ ኮሮና ውጤታማ ፈዋሽ ሕክምና የለም ፡፡ |  |  |  |
| 1. በዕድሜ የገፉ ወይም ሥር የሰደደ በሽታ ያላቸው ብቻ ለኮሮና በሽታ የተጋለጡ ናቸው ፡፡ |  |  |  |
| 1. ኮሮና በ ዋናነት የሚሰራጨዉ በንክኪ ነዉ፡፡ |  |  |  |
| 1. የ ኮሮና ምልክቶች የሌሉባቸው ሰዎች ቫይረሱን ለሌሎች ማስተላለፍ ይችላሉ፡ |  |  |  |
| 1. ኮሮና በሽታ ያላቸው ሰዎች ትኩሳት በማይኖርበት ጊዜ ቫይረሱን ለሌሎች ማሰራጨት አይችሉም ፡፡ |  |  |  |
| 1. የዱር እንስሳትን መብላት ወይም መንካት የ ኮሮና ቫይረስን ያስከትላል ፡፡ |  |  |  |
| 1. ለቫይረሱ ከተጋለጡ እስከ ምልክቶቹ መጀመሪያ ድረስ ያለው ጊዜ ከ2-14 ቀናት ነው |  |  |  |
| 1. ጭምብል መልበስ በ ኮሮና ቫይረስ እንዳንጠቃ ይከላከላል |  |  |  |
| 1. በ ኮሮና ቫይረስ የተያዙ ሰዎችን በተገቢው ቦታ መነጠል እና ህክምና የቫይረሱን ስርጭት ለመቀነስ ውጤታማ መንገዶች ናቸው ፡፡ |  |  |  |
| 1. እጅን ቢያንስ ለ 20 ሰከንዶች በሳሙና እና በውኃ ማጠብ ኮሮና ን ለመከላከል አንዱ ዘዴ ነው ፡፡ |  |  |  |
| 1. ከቤትዎ ውጭ ያሉትን ሁሉንም ነገሮች ከነኩ በፊት እና በኋላ እጅን መታጠብ ያስፈልጋል ፡፡ |  |  |  |
| 1. ከግለሰቦች በ 2 ሜትር ርቀት መኖሩ የ ኮሮና መተላለፍ አደጋን ሊቀንስ ይችላል፡፡ |  |  |  |

ክፍል 3: የጥናቱ ተሳታፉዎችን ስለ ኮሮና በሽታ ያላቸዉን አመለካከትለመለካት የተዘጋጀ መጠይቅ (ትክክለኛዉ መልስ ላይ የ ‘’X’’ ምልክት ይደረግ)

| መጠይቅ | በጣም አልስማማም | አልስማማም | ገለልተኛ | እስማማለሁ | በጣም እስማማለሁ |
| --- | --- | --- | --- | --- | --- |
| - - - 1. ኮሮና ቫይረስ ከባድ በሽታ ነው ፡፡ |  |  |  |  |  |
| - - - 1. የ ፊት ጭምብልን በ ጥሩ ሁኔታ መልበስ ኮሮና ቫይረስን ለመከላከል ውጤታማ ነው ፡፡ |  |  |  |  |  |
| - - - 1. እጅን መታጠብ ከ ኮሮና ለመጠበቅ ይጠቅማል |  |  |  |  |  |
| - - - 1. ኮሮና ቫይረስ መገለል ስላልሆነ አልደብቅም |  |  |  |  |  |
| - - - 1. ኮሮና ቫይረስ ን ለመቆጣጠር የራስ-ጥረት ውጤታማ ነው |  |  |  |  |  |
| - - - 1. ከ ኮሮና ቫይረስ ራሴን መከላከል እንደምችል አምናለሁ ፡፡ |  |  |  |  |  |
| - - - 1. ወደ የተጨናነቀ ቦታ መሄድ እና ከብዙ ሰዎች ጋር መገናኘት በ ኮሮና ቫይረስ የመያዝ አደጋን ያስከትላል |  |  |  |  |  |
| - - - 1. በ ኮሮና ቫይረስ እንዳልያዝ ስለምሰጋ ብታመምም እንኩዋን ወደ ሆስፒታል አልሄድም |  |  |  |  |  |
| - - - 1. ኮሮና ቫይረስ ን በመጨረሻ በተሳካ ሁኔታ እንቆጣጠረዋለን ብዬ አምናለሁ |  |  |  |  |  |
| - - - 1. ቫይረሱን ለመለየት የሚደረግ ላቦራቶሪ ምርመራ ካለ ለማድረግ ዝግጁ ነኝ፡፡ |  |  |  |  |  |
| - - - 1. ለ ኮሮና ቫይረስ የሚሆን ክትባት ካለ ለመዉሰድ ዝግጁ ነኝ፡፡ |  |  |  |  |  |

| ክፍል 4 የአፍና አፍንጫ ጭንብል ከመልበስ ባህሪያት ጋር የተያያዙ ጥያቄዎች | |
| --- | --- |
| 1. የጥናቱ ተሳታፊ በ ቃለ መጠይቅ ጊዜ የአፍና አፍንጫ ጭንብል ለብሰዋል? (በምልከታ የሚመለስ) | 0. የለም 1. አዎ |
| 1. የአፍና አፍንጫ ጭንብሉ የትኛዉን የፊት ክፍል ይሸፍናል? (በምልከታ የሚመለስ) | 1. አፍና አፍንጫን 2. አፍ፣አፍንጫ፣አገጭ እና የፊት ላይ ፀጉር 3. ሌላ ይገለጽ_____________ |
| 3. በሥራ ላይ እያሉ ምን ያህል ጊዜ ጭምብል ይለብሳሉ? | 1. ሁል ጊዜ  2. አንዳንድ ጊዜ  3. በጭራሽ አለብስም |
| 4. ምን ዓይነት ጭምብል ይመርጣሉ? | 1. የሕክምና የአፍና አፍንጫ ጭንብል  2. የጨርቅ የአፍና አፍንጫ ጭንብል  3. N95 የአፍና አፍንጫ ጭንብል  4. ሌላ (ይግለጹ) _________ |
| 5. ጭምብልዎን ምን ያህል ጊዜ ከተጠቀሙ በኃላ ይለውጣሉ? | 1. ለ 2-4 ሰዓታት  2. 1 ቀን  3. 2-5 ቀናት  4. ከ 5 ቀናት በላይ  5. አዲስ ጭምብል ሳይተኩ ፣ካጸዱ በኋላ መጠቀሙን መቀጠል |
| 6. ትክክለኛዉ የሕክምና የአፍና አፍንጫ ጭንብል አለባበስ እንደት ነዉ? (ትክክለኛዉን የዉጭና ዉስጥ፤ እንዲሁም የላይና ታች አቀማመጥ ለዩ) | |
| - 1. ጭምብሉ ውስጡ ነጣ እና ውጩ ጠቆር ያለ ነው ፣ እና ከላይ የብረት ማሰሪያዎች (የአፍንጫ ክሊፖች) አሉት | 0. የለም 1. አዎ |
| 6.2 አፍንጫውን ፣ አፍን እና አገጭውን ለመሸፈን ጭምብሉን የታጠፈውን ፊት ሙሉ በሙሉ ይበትናሉ? | 0. የለም 1. አዎ |
| 6.3 የሁለቱን እጆች ጣቶች በአፍንጫ ክሊፕ ላይ አድርገዉ ፣ ከመካከለኛው ቦታ ጀምረዉ እና በአፍንጫው ድልድይ ቅርፅ መሠረት የአፍንጫውን ክሊፕ ቅርጽ ያስይዛሉ ፡፡ | 0. የለም 1. አዎ |
| - 1. የጭምብሉ ጠርዝ ከፊትዎ ጋር የሚስማማ መሆኑን ያረጋግጣሉ | 0. የለም 1. አዎ |
| 6.5 የፊትና የኋላ ክፍል ሳይለዩ ይለብሳሉ | 0. የለም 1. አዎ |
| 6.6 የሕክምና የአፍና አፍንጫ ጭንብል በጭራሽ አልጠቀምም | 0. የለም 1. አዎ |
| 7. የአፍና አፍንጫ ጭንብል ካደረጉ በኃላ የትኛዉን ተግባር ይፈፅማሉ (ከአንድ በላይ መልስ ይቻላል) | 1.የጭንብሉን ፊተኛዉን ክፍል በእጅ መንካት  2. የራስዎን ወይም የሌሎችን ጭምብል በቀላሉ ለማንሳት ነፃነት መሰማት  3. ጭምብሉን አውልቀው መልሰው መልበስ  4. አፍንጫውን ወይም አፍን ለማሳየት ጭምብሉን ወደ ታች መጎተት  5.ሁሉንም ከላይ ያሉትን ባህሪዎች አልተገብርም |
| 1. የፊት ጭምብል ማድረጉ በስራዎ ላይ ምቾት አለው? | 0. የለም 1. አዎ |
| 1. የፊት ጭንብል ማድረግ በንግግር ጊዜ ምቾት ይነሳልን? | 0. የለም 1. አዎ |
| 1. የፊት ጭንብል በሚያደርጉ ጊዜ ፊት ላይ አሻራ ይኖረዋል | 0. የለም 1. አዎ |
| 1. የጭንብልወን ንጽህና እንደት የጠብቃሉ (እንደገና ጥቅም ላይ ለሚዉሉ ጭምብሎች ብቻ | 1. በዉሀ ብቻ በማጠብ 2. በሙቅ ዉሃና በሳሙና አጥቦ ፀሀይ ላይ ለ 5ሰአት በማድረቅ 3. ለ 5 ደቂቃ ማጠብ እና መቀቀል ከዛ ማድረቅ 4. ጭምብሉን በየቀኑ ለ 5 ደቂቃ መተኮስ 5. ሌላ (ይገለፅ) ___________ |
| 1. የፊት መሸፈኛ ጭምብልን ከተጠቀሙ በኋላ በምን ይሰበስባሉ? (በምልከታ የሚመለስ) | 1. በፔዳል የሚሰራ የቆሻሻ ማጠራቀሚያዎች 2. መሸፈኛ ክዳን ያለዉ የቆሻሻ ማጠራቀሚያ 3. ክዳን የሌለዉ የቆሻሻ ማጠራቀሚያ 4. ሌላ (ይገለፅ) ___________ |
| 1. የተሰበሰበዉን ቆሻሻ እንደት ያስወግዳሉ? | 1. ክፍት የሆነ ቦታ ላይ መጣል 2. ማቃጠል 3. በማዘጋጃ ማስነሳት 4. ሌላ (ይገለፅ) ___________ |
| ክፍል 5 ጭምብል መልበስ እና የእጅ ንፅህና አጠባበቅ ባህሪያትን የሚወስኑ ምክንያቶች ጋር የተያያዙ ጥያቄዎች | |
| 1. ለ ኮሮና በሽታ ተጋላጭ እንደሆኑ ይሰማዎታል? | 0. የለም 1. አዎ |
| 2. የ ኮሮና በሽታን ይፈራሉ? | 0. የለም 1. አዎ |
| 1. በ ኮሮና በሽታ መያዝ የሚያስከትለው መዘዝ ከባድ እንደሆነ ይሰማዎታል? | 0. የለም 1. አዎ |
| 1. ለ ኮሮና ቫይረስ የምርመራ ውጤቱ ፖዘቲቭ የሆነ ሰው ያውቃሉ? | 0. የለም 1. አዎ |
| 1. በ ኮሮና ቫይረስ የሞተ ሰው ያውቃሉ? | 0. የለም 1. አዎ |
| 1. ከሀገር ዉጭ ተጉዘዉ ያዉቃሉን | 0. የለም 1. አዎ |
| 1. ብር ለመቁጠር ምን ዘዴ ይጠቀማሉ | 1.ምራቅን በመጠቀም  2. ብር መቁጠሪያ ኬሚካልን በመጠቀም  3. ዉሃ በመጠቀም  4. ሌላ ይገለፅ­­­­­­­­­­­­­­­­­­_____________ |
| 1. ከላይ ለተጠቀሰው ጥያቄ የሚሰጠው መልስ ምራቅ በመጠቀም ከሆነ በምላስዎ እና ጣቶች መካከል ንክኪ አለን? | 0. የለም 1. አዎ |
| 1. ከሥራ ባልደረቦችዎ 6 ጫማ (2 ሜትር) አካላዊ ርቀትን ይጠብቃሉ? | 0. የለም 1. አዎ |
| 1. ከደንበኛ 6 ጫማ (2 ሜትር) አካላዊ ርቀትን ይጠብቃሉ? | 0. የለም 1. አዎ |
| 11. ሲጋራ ያጨሳሉን | 0. የለም 1. አዎ |
| 12. የአልኮል መጠጥ ይጠጣሉን | 0. የለም 1. አዎ |
| 13. እንደ sinusitis ያሉ የመተንፈሻ አካላት ችግር አለብዎ | 0. የለም 1. አዎ |
| 1. እንደ የደም ግፊት ፣ የስኳር በሽታ ያለ ሥር የሰደደ በሽታ አለብዎ | 0. የለም 1. አዎ |
| 1. ስለ COVID-19 የጤና መረጃ ያገኛሉ | 0. የለም 1. አዎ |
| 1. መልስዎ አወ ከሆነ መረጃ ከየት ነዉ የምታገኘዉ/ኚዉ | 1. ከዜና ሚዲያ ለምሳሌ ፣ ቴሌቪዥን ፣ ሬዲዮ ፣ ጋዜጣ  2. ከጤና ባሇሞያዎች  3. ማህበራዊ ሚዲያ  4. ከ ጓደኞች  5. ሌላ (ይገለፅ)__________ |
| 17. ጭምብል ስለ መልበስ እና እጅን መታጠብ የሚያሳይ ስዕላዊ መግለጫ አለ?(በምልከታ የሚመለስ) | 0. የለም 1. አዎ |
| 18.ኮሮናን በተመለከተ ስልተና ወስደዋልን |  |

ስለተሳትፎዎ በጣም አመሰግናለሁ!!!
