# Supplementary material for: Facemask-wearing behavior to prevent COVID-19 and associated factors among public and private bank workers in Ethiopia
Source: PLoS One. 2021 Dec 1;16(12):e0259659. doi: 10.1371/journal.pone.0259659 (PMC8635365; doi:10.1371/journal.pone.0259659)
Supplement: Attachment — Submitted filename: Response to Reviewers.docx [file pone.0259659.s004.docx]

**Line-by-line response to reviewers**

We would like to express our gratitude to the academic editor and both reviewers for their comments and ideas. We revised the manuscript and our responses are here below.

**For journal office requirements**

1. Please ensure that your manuscript meets PLOS ONE's style requirements, including those for file naming.

**Response: -** Thank you for this key comment. We formatted the manuscript according to PLOSE ONE author’s guidelines.

2. Please amend your current ethics statement to address the following concerns: Please explain why written consent was not obtained, how you recorded/documented participant consent, and if the ethics committees/IRBs approved this consent procedure."

**Response: -** Sorry for the confusion we created during writing the ethical statement. We already used written consent based on the decisions of ethical review board committee of Wollo University College of Medicine and Health Science.

3. Please include additional information regarding the survey or questionnaire used in the study and ensure that you have provided sufficient details that others could replicate the analyses. For instance, if you developed a questionnaire as part of this study and it is not under a copyright more restrictive than CC-BY, please include a copy, in both the original language and English, as Supporting Information. If the original language is written in non-Latin characters, for example Amharic, Chinese, or Korean, please use a file format that ensures these characters are visible."

**Response: -** Thank you for these key comments. As supporting information, we provided the questionnaire by original language and local language version.

4. Please state whether you validated the questionnaire prior to testing on study participants. Please provide details regarding the validation group within the methods section.

**Response: -** As discussed in the data quality assurance to keep the validity of the questionnaire, we developed the questionnaire from different published literature and COVID-19 related reports to make it more available. We did a pre-test for the questionnaire before data collection to improve the content of the questionnaire (Please see the revised version in page 9 from lines 186-189).

**Response to reviewer #1**

1. P4, line 76: there should be a period after the citation.

Response: - Sorry for this error. We accepted the comment and it’s corrected.

2. P5, line 89: I suggest to combine the paragraph beginning with “cases of COVID-19” with the first paragraph as these two paragraphs were more closely related, which introduced the situation of COVID-19 pandemic.

Response: Thank you and we updated accordingly. (You can see lines 93 for the improvement.)

3. The introduction should provide information about the preventive measures in the Dessie City. Many countries have implemented massive preventive measures to contain the spread of COVID-19 such as lockdown, social distancing, and face-masking wearing in all public venues. Any preventive measures in the investigated city? Is it mandatory? Such background information should be introduced to help readers understand the situation.

Response: Thank you for the very important comments. We included those preventive measures in the introductory part. (Please see in page 4 from lines 77-79 for the improvement.)

4. Any similar COVID-19 research among bank workers (e.g., the prevalence of COVID-19 infection or prevalence of preventive measures in this population)? I think the introduction should give more discussion about this specific population.

Response: Although we appreciate the comments, we are unable to find out any study on prevalence and preventive measures of COVID-19 in this study population before this study is conducted. We mentioned this gap in the background section of the last paragraphs (from line 109-111) and that is way this study was conducted.

5. P6, line 109: as this study was only conducted in the Dessie city, I suggest to describe the COVID-19 situation in Dessie city during the time of investigation.

Response: - Thank you; we described the situation of COVID-19 in Dessie city. (please see line 100 and 101 of page 5)

6. P7, line 31: what about the proportion of randomly selected participants from each back?

Response: - The numbers of workers were taken from attendance sheets in each selected branch by proportionally determining how much samples to be taken from each branch. After getting their number, samples were taken randomly from the sheet first by allocating the entire sample proportionally to the total number of workers in the selected bank branches using proportional allocation.

7. The author should provide more information about the measurements, e.g., the reliability of the used scales and whether these scales have been used in previous studies or they were self-constructed items. I suggest adding an appendix about the measurements as it was only briefly mentioned in the text.

Response: - we tried to elaborate the reliability of the questionnaire as well as the scales used in line with previous used studies as a reference in ‘Data collection procedures and quality assurance ’ part of the manuscript.

8. P13, line 277: there should be a period after the citation.

Response: - corrected

9. Did the participants know they would have a survey about face-masking in advance? This might have an influence on their face-masking behavior due to social desirability. The author may discuss this point.

Response: - They are told about the objective of the study therefore they know about face-masking since one objective is addressing face-mask wearing behavior of respondents, but we minimize social desirability bias by observing utilization of facemask at the time of data collection.

10. There are some spelling errors (e.g., p12 line 252, it should be "showed"). The author should have a careful check.

Response: - We corrected all these spelling errors

11. For discussion, the author should consider the local context and local COVID-19 situation to discuss and interpret the findings as the study was conducted in a single city. The generality of findings should also be discussed.

Response: - The reason for using COVID-19 situation at global level and study area other than bank was scarcity of COVID-19 related studies on the behavior of facemask wearing among bank workers at local level.

**For reviewer #2**

1. It is suggested further elaborating on the high risk of COVID-19 infection/transmission and thus needs for targeted health promotion among this population. Now the authors only mentioned that these workers have frequent direct or indirect contact with the public, however, this also applies to many other types of workers in the service industry. It is not clear how the findings would contribute to the extant literature.

Response: - Starting from the title the target of the study is on bank workers even if there are other sectors that have direct or indirect contact with many peoples. Additional reasons were also included in the manuscript.

2. Although the study focused on a special population group, the studied factors are quite general. To inform the development of tailored promotion strategies among this population, more bank worker-specific factors should be included, such as some contextual factors regarding protection measures provided in the working environment. It
   shall be mentioned somewhere in the Discussion that how the identified relationships are different from/consistent with those found among general population or other working population.

Response: - Despite the fact that general factors were included in the research, this study also included specific factors to analyze the bank environment and bank personnel.

3. Now the measures of knowledge and attitudes were dichotomized for analysis based on the mean score of the sample rather than a more valid and meaningful cut-off point. I suggest treating them as the original continuous variables as dichotomization itself caused loss of information and reporting the reliability alpha for the attitude scale
   as well. Also, it is not suggested defining those with higher scores as “positive attitudes” and those with lower scores as “negative attitudes”, as the valence of attitude shall be inherently determined by the measurement items, for example, there are both positive and negative attitudes toward mask wearing.

Response: - The reason for using mean for categorizing continuous variables of knowledge and attitude is to get appropriate number of responses. And this method was used by other several published COVID-19 papers. The other reason for using the mean is when other way of classification was applied we faced less observation which can’t allow as for further analysis.

4. The study explored the use of “non-medical mask” which is good, but please clarifies a bit whether the measure of “facemask wearing behavior” captured this aspect when defining good behavior of facemask wearing. Please also clarify that whether this behavior was self-reported or objectively observed by the interview fieldworkers.

Response: - Our criteria for saying good/poor behaviors of face mask wearing; not include type of face mask used as operationally defined, but this descriptive data were collected based on self-reported data since there were respondents who don’t were face mask at the time of data collection.

5. The study used “cluster random sampling” but not “simple random sampling”, please revise the related parts accordingly. The selected banks shall be included as a covariate in the analysis or multilevel regression models shall be used for handling such clustered data.

Response: -There was no clustering applied in this study, the only reason for saying government and private bank was to describe and see the difference if any among the two. Like other variables used which were expected to be a factor or not. Simply we select 50% of the bank branches randomly either from government or private and the workers were selected by proportionally allocating their number in that selected branch.

6. Table 1-5 shall be combined into one table to make the presentation more succinct.

Response: -If we merge the 5 tables as one Table, it becomes very large and bulky to understand it.

**We would be happy to make further corrections if necessary and look forward to hearing from you all soon.**

I hope that the revised manuscript is accepted for publication in PLoS ONE.
