# Supplementary material for: Facemask-wearing behavior to prevent COVID-19 and associated factors among public and private bank workers in Ethiopia
Source: PLoS One. 2021 Dec 1;16(12):e0259659. doi: 10.1371/journal.pone.0259659 (PMC8635365; doi:10.1371/journal.pone.0259659)
Supplement: Attachment — Submitted filename: Response to Reviewers second.docx [file pone.0259659.s005.docx]

**Response to editor and reviewer #2**

We would like to express our gratitude to the academic editor and both reviewers for their comments and ideas for the first revision. We revised the manuscript for the second time and our responses are here below.

**Additional editor comments**

1. “I noticed that some comments raised by reviewer 2 were not addresses during the revision. The author should address all comments raised by the reviewers”

**Response: -** Thank you for this main comment for second revision. We see all the comments that were not seen.

**For reviewer #2**

1. First, please discuss the generalizability of the findings to general population and other work population. Please refer to my previous #2 comment.

**Response:** - Since the study includes both variables for the general population and specific variables for bank workers we can show the generalizability of the study can be for bank workers as well as for the general population. This generalizability of the study was indicated in the manuscript, line 375-378 of page 17.

2. Second, regarding my previous #3 comment, 1) I suggest not defining those with higher scores as “positive attitude” and those with lower scores as “negative attitude”. Instead, high/low level of attitudes can be used; 2) please report the reliability alpha for the attitude scale. This is necessary even if a dichotomized form was used for analysis.

**Response: -** It was corrected as high/low level of attitude, changed in all parts in the manuscript and Cronbach alpha or reliability alpha coefficient for attitude was 0.87.

3. Third, regarding my previous #4 comment, necessary information has been provided by the authors, please also include it in the manuscript, e.g., the measure of facemask wearing behavior” captured whether wearing a facemask of any type; and it is a self-reported measure.

**Response: -** Thank you for all your comments and suggestion, it was indicated in line 197-204, page 9&10 of the manuscript.

**We would be happy to make further corrections if necessary and look forward to hearing from you all soon.**

I hope that the revised manuscript is accepted for publication in PLoS ONE.
